# Combinatorial Epigenetics Impact of Polyphenols and Phytochemicals in Cancer Prevention and Therapy

**DOI:** 10.3390/ijms20184567

**Published:** 2019-09-14

**Authors:** Itika Arora, Manvi Sharma, Trygve O. Tollefsbol

**Affiliations:** 1 Department of Biology, University of Alabama at Birmingham, 1300 University Boulevard, Birmingham, AL 35294, USA; itiarora@uab.edu (I.A.); manvi@uab.edu (M.S.); 2 Comprehensive Center for Healthy Aging, University of Alabama Birmingham, 1530 3rd Avenue South, Birmingham, AL 35294, USA; 3 Comprehensive Cancer Center, University of Alabama Birmingham, 1802 6th Avenue South, Birmingham, AL 35294, USA; 4 Nutrition Obesity Research Center, University of Alabama Birmingham, 1675 University Boulevard, Birmingham, AL 35294, USA; 5 Comprehensive Diabetes Center, University of Alabama Birmingham, 1825 University Boulevard, Birmingham, AL 35294, USA

**Keywords:** epigenetics, cancer, polyphenols, DNA methylation, histone modifications, microRNA

## Abstract

Polyphenols are potent micronutrients that can be found in large quantities in various food sources and spices. These compounds, also known as phenolics due to their phenolic structure, play a vital nutrient-based role in the prevention of various diseases such as diabetes, cardiovascular diseases, neurodegenerative diseases, liver disease, and cancers. However, the function of polyphenols in disease prevention and therapy depends on their dietary consumption and biological properties. According to American Cancer Society statistics, there will be an expected rise of 23.6 million new cancer cases by 2030. Due to the severity of the increased risk, it is important to evaluate various preventive measures associated with cancer. Relatively recently, numerous studies have indicated that various dietary polyphenols and phytochemicals possess properties of modifying epigenetic mechanisms that modulate gene expression resulting in regulation of cancer. These polyphenols and phytochemicals, when administrated in a dose-dependent and combinatorial-based manner, can have an enhanced effect on epigenetic changes, which play a crucial role in cancer prevention and therapy. Hence, this review will focus on the mechanisms of combined polyphenols and phytochemicals that can impact various epigenetic modifications such as DNA methylation and histone modifications as well as regulation of non-coding miRNAs expression for treatment and prevention of various types of cancer.

## 1. Introduction

Cancer is the second leading cause of death in the United States [1]. Globally, 9.6 million deaths occurred in the year 2018 compared to 7.6 million cancer-associated deaths in 2008. Collectively, cancer is comprised of a heterogeneous group of disorders which involves uncontrolled proliferation of previously healthy cells [2]. Even though there are over 100 different types of cancers, the primary contributors to the increased mortality rates are cancer of the breast, lung, prostate, colon and rectum (a.k.a. colorectal cancer) [3,4,5]. It is, therefore, imperative to evaluate the factors leading to different types of cancers, their prevention, and therapeutic measures. The uncontrolled proliferation of cancer cells originates locally and may widely spread through metastasis. This process further results in loss of control of cell growth, eventually leading to the invasion of cancer cells to healthy tissues [6]. 

Factors such as alcohol consumption, exposure to harmful chemicals due to smoking, an unhealthy diet, and physical sedentariness result in higher rates of lifestyle-associated cancer risks (cancer-transition) in part by inducing mutations in DNA. Genetic changes and epigenetic aberrations play a critical role in the progression of the disease and each of these alterations is known to be an essential hallmark for different types of cancer [7,8,9,10]. Traditionally, cancer research was primarily focused on genetic changes, mainly elucidating the overexpression/mutation of oncogenes and inactivation of tumor suppressor genes (TSGs). Each of these changes reinforces major cancer pathways such as the RTK/RAS pathway, PI3K pathway, Wnt pathway, Myc pathway, p53 pathway, Notch Signaling pathway, Nrf2 pathway, and cell cycle pathways [11,12,13,14,15,16]. Since the 1990s, cancer research has also centered around understanding heritable changes which regulate various epigenetic alterations. Therefore, it is crucial to understand the etiology behind epigenetics mechanisms, which eventually lead to carcinogenesis [17]. Epigenetics is the study of changes in the phenotypes that do not arise from alterations in the DNA sequence. The various epigenetic mechanisms include DNA methylation, histone tail modifications, non-coding RNA functions, regulation of polycomb assembly proteins, and cofactor modifiers. DNA methylation and histone modifications are the primary contributors to cancer epigenetics machinery that eventually may cause alterations in gene expression but no alteration in the DNA sequence [18,19,20,21,22]. Unlike genetic changes such as mutations and deletions, which can be difficult to reverse [23,24], epigenetics aberrations are often reversible. Epigenetically altered genes can be potentially corrected, by reversing the alteration in DNA methylation found in CpG dinucleotides, for example, thereby causing transcriptional activation of histone complexes by acetylation and methylation [25]. Epigenetic modifications are also known to be a dynamic hallmark of cancer due to their massive impact on cell proliferation and differentiation. 

**Figure 1:**
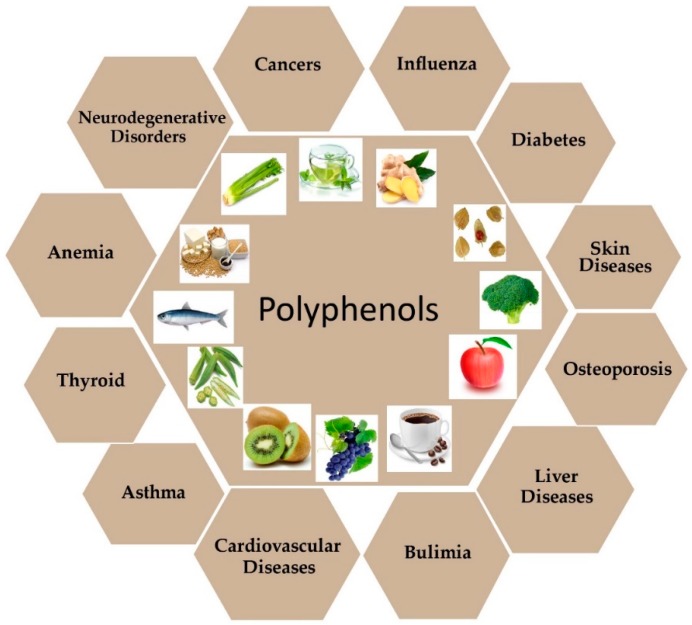
Health benefits effect of dietary polyphenols. Polyphenols are largely found in fruits, vegetables, spices, and beverages. Most of these compounds are involved in protection against the development of chronic diseases such as cardiovascular diseases (CVDs), neurodegenerative diseases, cancer, diabetes, osteoporosis, and liver diseases.

Various studies have reported strong evidence that natural compounds can potentially regulate gene expression by targeting different foundations/components of the epigenetic machinery [26]. With the continuous advancement of the lifestyle changes, it is necessary to extract new molecules, which can be potentially used for disease prevention and to find new drugs which may be crucial for cancer patient survival. The natural compounds are extracted in part from vegetal [27], microbial [28], and marine species [29] (Figure 1). Each of these can widely be used as a major source of activities against cancer and other diseases such as diabetes [30], cardiovascular diseases [31], liver diseases [32], asthma [33], neurodegenerative diseases [34], osteoporosis [35], anemia [36], bulimia [37], influenza/ pneumonia [38], renal and thyroid disorders [39,40], nephritis [41], periodontal (gum) disease [42], hypertension [43] and skin disorders [44] (Figure 1). 

Amongst various natural compounds, polyphenols have predominantly evolved as a large group of compounds by providing resistance and immunity against ultraviolet exposure, signal transduction and host defense against pathogens [45]. Although polyphenols are primarily involved with numerous mechanisms, through interactions with various cellular components such as carbohydrates, proteins and enzymes for the regulation of gene expression, they also exhibit active involvement in cancer pathways, in particular, signaling pathways [46,47]. Plant-based polyphenols are well-known to modulate cancer pathways by inhibiting cancer cell proliferation, which can cause an overall decrease in tumor mass, thus allowing tumor regression. 

In spite of large preventive measures, plant-based dietary polyphenols also exhibit a significant role in protecting the healthy cells from adverse effects of chemotherapy by enhancing the cytotoxic activity of chemotherapeutic agents in cancerous cells [48]. 

A plethora of studies have described the anticancer mechanisms of polyphenols for individual compounds [49]. Despite being potent anti-cancer agents, many polyphenols have poor bioavailability thereby impeding there in vivo effects, mainly when used individually [50]. Their scope of efficacy can be increased by combining them with other different polyphenols and phytochemicals for potential synergistic effects. Here, we will focus on the combinatorial effects of various polyphenols, phytochemicals, and anti-cancer drugs on the epigenetics machinery by providing insights into their specific epigenetics targets associated with cancer prevention and therapy. 

## 2. Dietary-Based Polyphenols: Role in Cancer Prevention and Therapy

Many studies have demonstrated the use of plant or animal-based natural compounds for prevention and treatment of multiple diseases such as asthma, cardiovascular diseases, pathogens protection, diabetes, neurodegenerative diseases and cancer [51]. More than 8000 polyphenolic compounds are from plant species [52]. Multiple studies have shown that some plant-based polyphenols possess anti-cancerous properties such as inhibition of cell proliferation, tumor growth, angiogenesis, metastasis, inflammation, and apoptosis [47,50]. These polyphenols can also be used as active compounds to develop novel chemopreventive agents, which can be highly effective while conferring little if any toxicity [47]. 

**Figure 2:**
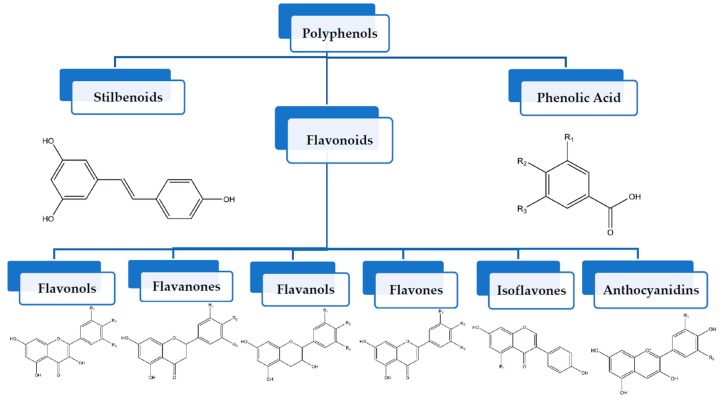
Different classification of polyphenols and their chemical structures. Flavonoids are subdivided into flavonols, flavanones, flavanols, flavones, isoflavones, and anthocyanidins.

Polyphenols can be broadly classified into three main categories; flavonoids, stilbenoids, and phenolic acids [53] (Figure 2). 

Among these, flavonoids are the largest group that are comprised of approximately 5000 polyphenols [54]. These classifications differ from each other based on the number of phenolic rings and their structural elements. Flavonoids account for about 60% of polyphenols which include two or more aromatic rings, linked by a carbon bridge containing three carbon atoms and the aromatic rings which possess one or more phenolic hydroxyl groups [55]. Flavonoids are subdivided into different subclasses: flavones, isoflavones, flavonols, flavanones, flavanols and anthocyanidins. Flavonoids possess various antioxidant and anti-inflammatory properties [56]. Among these, flavanols are the most abundant and are found in different food sources. For instance, quercetin, curcumin (CUR), and epigallocatechin-3-gallate (EGCG) are bioactive flavonoid compounds found in black tea, turmeric, and green tea [57,58,59]. The flavanols are further categorized into monomers (such as catechins found in red wine and chocolate) and polymers (such as proanthocyanidins and theaflavins) [47,60]. Unlike flavanols, flavones are less abundant in fruits and vegetables but found in parsley and celery to a greater extent [61]. Isoflavones, also known as phytoestrogens (due to their structural similarity to estrogens), are found in leguminous plants [62]. Table 1 provides a comprehensive list of the chemical structures and molecular formulas of the key polyphenols possessing anti-cancer properties.

Stilbenoids, another critical category of polyphenols, are a small group of compounds which contain polyhydroxystilbenes. These are found in lower quantities in our diet, thereby compromising their potential for significant health benefits. However, more massive amounts of stilbenoids can be provided from various strenuous extracts or as purified compounds. For instance, resveratrol (found in red wine, peanuts, grapes, and almonds) and pterostilbene (present in blueberries and grapes) are key stilbenoids that have anticarcinogenic properties and other health benefits [112]. Another primary classification of polyphenols, phenolic acids, account for 30% of polyphenols and consist of two main categories; hydroxybenzoic acid and hydroxycinnamic acid which are glycosylated derivatives of esters of quinic acid, shikimic acid, and tartaric acid. Hydroxybenzoic acid, is found in few consumable plants making it of lesser nutritional interest, although hydroxycinnamic acid is found in cinnamon, coffee, blueberries, kiwis, plums, apples, and cherries [113,114].

## 3. Bioavailability of Polyphenols

Bioavailability pertains to the process of nutrient digestion, absorption, and metabolism in biochemical pathways. After digestion and before absorption of polyphenols, they are hydrolyzed by intestinal enzymes that are present in the colon. During intake, polyphenols undergo various modifications and are further processed in the liver through methylation, sulfation, and glucuronidation [115]. It has been evident that polyphenols possess metabolic activities, which primarily depends on intrinsic activity, rate of metabolism, and their elimination. Polyphenol metabolic activity mainly occurs in the intestines and liver. Since most biologically active polyphenols are not very common in the diet, the bioavailability of polyphenols signifies a significant issue as they may reach the target organs in low concentrations [116]. Because of this many polyphenols portray a poor bioavailability as anti-cancer agents, thereby moderating in vivo effects.

One way to thwart this issue is with the help of nanotechnology, which plays a vital role in cancer prevention and treatment. Nanoparticle encapsulation of anticancer polyphenols can cause a several-fold increase to their oral bioavailability. For example, nanoformulations of curcumin and piperine combination led to a 9-fold expansion due to enhanced absorption thereby increasing efficacy and creating a dose advantage over free curcumin in different cancer cell lines [117]. Despite combating the low bioavailability of polyphenols, only a few combinations have been tried as nano-encapsulation as it can cause adverse side effects when administered in high doses [118,119,120]. Various studies have shown strong evidence of combinatorial effects of different polyphenols that increased chemoprotective and the anti-cancer properties at considerably lower concentrations [121]. This synergy of polyphenols in some cases is due to simultaneous impact on different cancer pathways as well as epigenetic modifications such as DNA methylation and histone modifications.

## 4. Epigenetics Mechanisms and Cancer

### 4.1. DNA Methylation

DNA methylation plays a crucial role in regulating growth and development of carcinogenesis by contributing to aberrations such as genomic instability, oncogenes activation and silencing of tumor suppressor genes (TSGs) which are mainly involved in cell proliferation, DNA repair and apoptosis [17,122,123,124]. The CpG positions are the areas of DNA where a cytosine nucleotide is followed by a guanine nucleotide in a 5′ → 3′ direction. These occur primarily in genomic as CpG islands. CpG dinucleotides are unevenly distributed in the human genomes but are common in promoter regions of genes. The DNA methylation state is modulated by the DNA methyltransferase (DNMT) enzymes. DNMTs are actively involved in the displacement of the methyl group from *S*-adenosyl-L-methionine (SAM) and placing it onto the 5-position of certain cytosines in CpG dinucleotides [49]. In mammals, there are three major types of DNMTs: DNMT1, DNMT3a, and DNMT3b [125]. DNMT1 is a ubiquitous enzyme and is primarily responsible for the maintenance of DNA methylation patterns during cell division. The DNMT3a and DNMT3b enzymes actively participate in *de novo* methylation processes, which involve the addition of a methyl group to cytosine and is necessary for differentiation [126,127].

Studies have provided strong evidence supporting the association of both DNA hypomethylation and hypermethylation sequences during cancer progression and have emphasized the importance of DNA hypomethylation and hypermethylation in the regulation of cancer-related genes [128]. The hypermethylation of CpGs can occur in the 5′ region of the cancer-associated genes and lead to inactivation of a significant number of tumor-suppressor genes during tumorigenesis in cancers. Either a single gene or small subsets of genes can be hypermethylated in different types of cancer [129]. For instance, *p16INK4a* (*CDKN2A*) which acts as a cyclin-dependent kinase inhibitor essential for TSGs, can undergo hypermethylation in cancer. Hypermethylation of TSGs such as *E-Cadherins* and *H-Cadherins* can cause metastasis, leading to tumor cells proliferation [130]. In addition, the silencing of *APC* gene has also been reported in various cancers such as breast, lung, prostate, and colorectal cancer. *APC* acts as an antagonist of the Wnt Signaling pathway, which is efficiently involved with cell migration and adhesion. Other instances of gene silencing are found in breast cancer, such as the silencing of *BRCA1*, resulting in DNA repair double-stranded breaks and transcription [131]. Unlike DNA hypermethylation of individual genes, genomic DNA hypomethylation of different genes in tumor cells is a rare phenomenon. DNA hypomethylation can cause chromosomal abnormality and induce mutations, thereby leading to activation of some transposable elements that may result in modification of the genome at random sites [132]. As a result, mutagenesis and genomic instability occur, thus leading to tumor development. Hypomethylation of *TTF-3* and *MUC4* frequently occurs in ovarian cancer, prostate cancer and pancreatic cancer [133].

A large number of FDA-approved drugs such as paclitaxel, doxorubicin, cisplatin, vorinostat, decitabine, and azacitidine have shown a potential role in cancer therapy via targeting the DNMTs. Therefore, the DNA methylation process is an important area with respect to epigenetic mechanisms leading to different types of cancer [134]. Only a few FDA-approved drugs have shown promising results in cancer patients due to their slight specificity towards cancerous cells [135]. Therefore, to increase the response of anti-cancer drugs towards cancerous cells, cancer patients are subjected to increased doses, which may result in adverse side effects.

Nutritional polyphenols and phytochemicals have an enormous impact on DNA methylation by causing changes in DNMTs levels via the direct or indirect effect on DNMT activity in cancer prevention and therapy. For instance, genistein of soy forms a complex with DNMT reducing methylation activity and resulting in activation of tumor suppressor genes which can eventually lead to cancer prevention and therapy [136]. Resveratrol, primarily found in grapes, also acts as a DNMT inhibitor, which may facilitate cancer prevention and treatment [137]. Table 2 provides a comprehensive list of different polyphenols and their effects on the DNA methylation epigenetic machinery.

### 4.2. Histone Modifications

Histones are soluble proteins involved in wrapping DNA into a structural unit called nucleosomes. The nucleosome, approximately ~146 bp, is positioned as beads at a regular distance [138]. Classically, a nucleosome is comprised of linker histones H1 and core histones: H2A/H2B, H3, and H4. Linker histones (H1) are also a primary component of nucleosomes [139]. The linker histone H1 binds to the outside of the nucleosome periphery and serves as a bridge between two adjacent nucleosomes. Core histones are more firmly bound to the DNA than H1 [140].

Histone modifications are also actively involved with tumor development and carcinogenesis [141] and most occur at the globular N-terminus domain which protrudes outwards from core histones H3 and H4. The N-terminus domain is prone to various chemical changes at lysine, serine, and threonine [142]. Post-translational modifications (PTMs) are also a primary component of the epigenome assembly which contributes to histone modifications. PTMs often lead to charge-induced changes in the nucleosome, which causes a massive influence on the gene expression. Histones associated with PTMs also assist many biological processes through chromatin modifications and PTMs can impact gene expression by altering chromatin structures, which contribute significantly to tumor development and carcinogenesis [142].

Even though PTMs are reversible, they are not restricted to lysine acetylation, lysine-arginine methylation, serine-threonine phosphorylation, and lysine ubiquitination [143]. Various catalytic enzymes such as histone acetyltransferases (HATs), histone deacetylases (HDACs), histone methyltransferases (HMTs) and histone demethylases (HDMs) also contribute to histone modifications. These induced histone modifications can result in cancer initiation and progression by causing genome-wide alterations [144]. HAT enzymes are actively involved in histone acetylation, which is responsible for the regulation of various cellular processes such as transcription, gene silencing, apoptosis, DNA repair, and cell differentiation [145,146]. Unlike HATs, HDACs are a class of enzymes which catalyzes the opposite action of HATs by influencing various processes such as signal transduction, apoptosis, and cell growth [147]. Histone acetylation imbalance due to these catalytic processes can lead to tumor cell development and cancer progression. HMTs and HDMs also act as a stimulus for histone modifications. HMTs are involved in DNA methylation via chromatin-dependent transcriptional repression and activation [148]. Due to these catalytic processes, specific genes within DNA complexed with histone can either be activated or silenced [149]. Amongst various types of HMTs, G9a and EZH2 are critical histone methyltransferases, as they catalyze methylation of histone H3 at lysine 27 (H3-K27). The H3-K27 methylation and lysine 9 histone H3 methylation (H3-K9) facilitate the development of heterochromatin resulting in gene silencing and contribute to cancer progression [150].

Many dietary polyphenols have promise in modulating histone modifications in cancer prevention and therapy. For example, sulforaphane, in broccoli, kale and cauliflower, complexes with the HDACs active sites thereby impeding HDAC activity [151].

### 4.3. Noncoding RNAs

Besides DNA methylation and histone modifications, microRNAs (miRNAs) also significantly contribute to epigenetic regulation. miRNAs can perform RNA splicing-related catalytic functions and miRNAs significantly contribute to post-translational gene regulations. miRNAs are small single-stranded non-coding RNAs that are 20–22 nucleotides long and regulate gene expression via post-translational silencing of the target genes [152]. miRNAs control numerous biological processes, such as cell proliferation, apoptosis, and cell differentiation. Due to their significant role in cell physiology, expression level alterations are directly related to disease progression. A large number of studies has shown direct association between miRNAs alterations and cancer [152,153,154,155]. MicroRNA expression can be regulated by different mechanisms such as chromosomal abnormalities, single nucleotide polymorphisms (SNPs), mutations in the primary transcripts such as *miR-15a* and *miR-16-1* [156], altered activity of different transcription factors such as *miR-17-92* cluster and changes in *miR-34* family due to activation of *p53*. These mechanisms can be associated with different types of cancers such those of the bladder, lung and breast [156,157,158]. For instance, hypermethylation of *miR-9-1* in breast cancer occurs while *miR-34b* and *miR-34c* clusters are hypermethylated in colorectal cancer [159,160]. Deviant methylation of *miR-9*, *miR-34b*, *miR-34c* and *miR-148a* are often associated with metastasis. Furthermore, methylation of *miR-148a*, *miR-34b/c* and *miR-9* are commonly associated with malignant cells [161]. In addition to these aberrations, promoter methylation and histone acetylation can also regulate microRNA expression in different types of cancer [162]. Table 2 provides a comprehensive list of polyphenols which are known to regulate epigenetic modifications associated with different types of cancer.

## 5. Combinatorial Effect of Polyphenols on Cancer Prevention and Therapy

A significant number of conventional methods are employed in cancer prevention or treatment. However, at some point, the tumor cells may develop resistance to various traditional methods such as radiotherapy and chemotherapy, thereby causing alterations in genes and proteins, which are involved in cancer progression. Therefore, combinatorial approaches can potentially be used in cancer prevention and therapy. These can be a combination of a polyphenol with two or more polyphenols, a combination of polyphenols with anti-cancer drugs, a combination of polyphenols with vitamin supplements or other efficacies in combination. These approaches can facilitate inhibition of tumor growth and in some cases the combined compounds can act synergistically. Here, we provide detailed information about various combinatorial approaches by different groups focusing on different types of cancer. These are summarized in the Table 3.

### 5.1. Combinatorial Effect of Apigenin with Other Polyphenols and Anti-Cancer Drugs

Many studies have reported various blockage in immune checkpoints that can lead to up-regulation of Interferon gamma (IFNγ) and further lead to tumor cell progression. A study was conducted demonstrating the combined action of apigenin and IFNγ. Primary cervical cancer HeLa and SiHa cells were co-administered with apigenin and IFNγ. This treatment resulted in enhancing the anticancer activity by targeting cyclin-dependent kinases 1. The HeLa and SiHa cells were treated with different doses of apigenin and IFNγ. As a result, it was found that HeLa cells were more sensitive than SiHa cells, and cell viability was further reduced with the treatment of apigenin when co-partnered with IFNγ. This combination also increased the upregulation of a number of tumor related genes. Furthermore, apigenin increased the apoptosis-inducing effects of IFNγ in HeLa cells but not in SiHa cells [289]. Paclitaxel is a chemotherapeutic FDA-approved drug used for the treatment of many different types of cancers such as ovarian, breast, lung, cervical and pancreatic cancer. Long-term administration of paclitaxel leads to the development of drug resistance and tumor progression. To overcome these, a combination of polyphenols such as apigenin can potentially be used. A study demonstrated the synergistic action of apigenin and paclitaxel in ovarian cancer. HeLa, A549, Hep3B, and HEK293A cells were treated with apigenin in combination with paclitaxel. As a result, both apigenin and paclitaxel induced apoptosis by eventually decreasing the number of surviving cells [290]. Cisplatin is a chemotherapeutic drug which is used in treating various cancers [291] and apigenin was also shown to amend cisplatin function in these cases. In human renal proximal tubular epithelial cells (HK-2cells), the combination of apigenin with cisplatin led to the reduction of p53 activation and further promoted the PI3K/Akt pathway. A study in prostate cancer in PC-3 cells and CSCs demonstrated a combined effect of apigenin and cisplatin by suppressing PI3K/AKT activation and protein expression of NF-κB [292,293].

Additionally, the combination of apigenin with doxorubicin induced a synergistic decrease in ATP levels in leukemia CCRF-CEM, Jurkat, and THP-1 cell lines. As a result, co-administration of apigenin and doxorubicin led to a decrease in ATP levels in three (CCRF-CEM, Jurkat and THP-1) out of four leukemia cell lines through enhancement in cell toxicity and DNA damage. This combination treatment also led to an increase in caspase-3 activity in all the four cell lines as well as cell cycle arrest and S and G2/M phase inhibition. Additionally, the combinatorial effect of apigenin and etoposide resulted in a decrease in ATP levels in the leukemia THP-1 myeloid cell line along with additive effects on other cell lines [294]. Another study was conducted in vitro in young adult mouse colonocyte cells (YMAC) to investigate the synergistic action of the two polyphenols. Higher concentrations of naringenin (5 µM and 10 µM) eradicated the growth of the cells, unlike apigenin, which abolished growth of the YMAC cells at a much lower concentration. However, the combination of apigenin and naringenin led to inhibition of YMAC cell growth, thereby causing activation of the estrogen receptor (ERβ) at a much lower concentration (0.1 + 0.05 − 1 µM) [295]. In pancreatic cancer, apigenin along with gemcitabine enhanced anti-tumor effects. In vitro, this combined treatment of apigenin and gemcitabine led to a decrease in tumor cell growth and apoptosis by down-regulating NF-kappa B activity. The combination also resulted in the suppression of Akt activation in MiaPaca-2 and APC-1 cell lines. Collectively, these combinatorial studies have demonstrated a strong impact in different types of cancer and their associated molecular mechanisms [296].

### 5.2. Combinatorial Effect of Curcumin with Other Polyphenols and Anti-Cancer Drugs

Primary prostate cancer cells in B6C3F1/J mice when treated with the combination of curcumin and resveratrol increased the bioavailability by decreasing the tumor growth and inhibition of epithelial cell proliferation in contrast to curcumin administered alone. The combination of curcumin and resveratrol reduced prostate cancer by controlling the mean GU tract and decreasing the tumor weight of the mice [297]. Another study was conducted in vivo on the 1, 2-dimethylhydrazine (DMH) rat model in colorectal tumors and demonstrated the combinatorial effect on curcumin and catechins. The dietary treatment of curcumin, catechins and the combination of curcumin and catechin were administered in the positive and treated groups. As a result, although the incidence of the colorectal tumor was lower in the catechins and curcumin treatment groups, the difference was not significant when compared to the treatment groups when catechins and curcumin were administered alone. However, the frequency of the colorectal tumor was significantly lower in the combination group when compared to the positive groups. The study also demonstrated that the cell proliferation index (PI) was more significantly inhibited with the combination group (PI index: 24.2 ± 9.02, *p* < 0.01) compared to the curcumin (PI index: 39.2 ± 7.26, *p* < 0.05) and catechin (PI index: 36.8 ± 5.50, *p* < 0.05) alone [298].

Another study in breast cancer (MCF-7 and Sum149 cells) demonstrated an improved bioavailability of curcumin and piperine in combination. Piperine (1-Piperoylpiperidine) is a dietary alkaloid which is mainly found in fruits and roots of black pepper [299]. This combination was known to be effective in cancer prevention by limiting stem cell self-renewal and inhibition of the Wnt signaling pathway. As a result, both curcumin and piperine inhibited mammosphere formation and serial passaging but the effect of inhibition was greater when both polyphenols were combined [300]. Curcumin also has positive effects with many other conventional therapies in breast cancer cells both in vitro and in vivo. In vitro, breast cancer MDA-MB-231 cells were more prone to inhibition by paclitaxel when combined with curcumin. In vivo, the 8-week-old athymic mice were administered with curcumin (100 mg/kg daily), paclitaxel (7 mg/kg weekly) and curcumin + paclitaxel. As a result, the combination treatment inhibited the growth of the cells to a more considerable extent compared to curcumin and paclitaxel alone. Eventually, curcumin inhibited the activity of NF-κB induced by paclitaxel, thereby increasing the apoptotic effect of paclitaxel [301].

Arcitgenin is a soluble plant extract of Arctium lappa which is used in Japanese Kampo medicine. Flavonoids such as curcumin and EGCG have reduced bioavailability when administered alone. Therefore, to overcome the reduced bioavailability of specific flavonoids, another study was conducted in breast cancer MCF-7 cells and prostate cancer LNCaP cells by administering a combination for 48 h. As a result, both cells lines demonstrated a synergistic increment of antiproliferative effect. In MCF-7 cells, arctigenin increased the cell apoptosis of curcumin and EGCG enhanced the cell cycle arrest of curcumin. This combination also led to an increased expression of Bax-Bcl2 proteins. Another study was conducted on non-small lung cancer (NSLC) A549 and NCI-H460 cells with the combination of low concentration of EGCG and curcumin. EGCG, when combined with curcumin, reduced the clonal formation in A549 cells. This combination heightened cell cycle arrest at G1 and S/G2 phase and inhibited cyclin D1 and cyclin B1. There was also a decrement in the tumor growth, thereby being a strong chemopreventive agent in NSLC. Also, the combination of curcumin (50 μM) and EGCG (100 μM) had a synergistic effect on prostate cancer LNCaP, DU145 and PC3 cells by causing the increased expression of p21, and cell cycle arrest at S and G2/M phase [302,303,304,305,306].

Primary colon cancer cells are commonly treated with the drug dasatinib, which is a small molecule-inhibitor of the SRC-family of protein kinases. Sustained chemotherapeutic treatment with this drug results in drug resistance and tumor progression. Therefore, to prevent these harmful effects, dasatinib when combined with curcumin using in vitro and in vivo models, resulted in the enhanced inhibition of various metastatic processes. In vivo, this combination enhanced the cell adhesion phenotype of colon cancer HCT-116 cells. In vitro, this combination led to a 95% regression of intestinal adenomas in APC^Min+/−^ mice, thereby decreasing tumor proliferation and increasing apoptosis [306]. TNF-related apoptosis-inducing ligand (TRAIL) is a tumor necrosis factor (TNF) gene which possesses apoptosis-inducing activity against cancer cells in vivo and in vitro. Despite this functionality, the defects in intrinsic and extrinsic pathways (such as Akt and NF-κB pathways) could potentially cause cell resistance thereby affecting its functional role. Thus far, the combinatorial approach of TRAIL with curcumin can cause synergistic action in prostate cancer PC3 cells. This combination caused suppression of NF-κB activity through Akt pathways, and further inhibition of Bcl-2, Bcl-XL, and XIAP expression. In vivo, when this was administered to xenografted mice with prostate LNCAP cells, the inhibition of tumor growth, increased apoptotic activity, and further activation of anti-proliferative, anti-angiogenic, and anti-metastatic mechanisms were observed [307,308]. Gemcitabine is a chemotherapeutic drug which is known to treat various types of cancer such as those of bladder, pancreatic and breast. Like Dasatinib, this drug also results in drug resistance. When gemcitabine (25 mg/kg body weight once every four weeks) is used in combination with curcumin (100 mg/kg body weight daily) in pancreatic cancer BxPC3, MiaPaCa2 and Panc1 PDAC cells, it inhibited tumor growth thereby inhibiting expression of PRC2 subunit EZH2 and lncRNA *PVT1*. This combination also suppressed the spheroid-forming capability of tumor cells [309].

### 5.3. Combinatorial Effect of Genistein with Other Polyphenols and Anti-Cancer Drugs

The primary mechanism of genistein is to induce DNA strand breaks and oxidative stress. On the other hand, delphinidin suppresses DNA-damaging properties and possess anti-oxidative properties. Despite possessing strong anti-cancer properties, their systemic bioavailability is low. Therefore, both genistein and delphinidin, when used in combination with alternariol (AOH) which is predominantly found in mushrooms, strongly interact with cancer cells. This combination demonstrated strong interactions with the HT-29 colon carcinoma cells and by influencing topoisomerase poisoning and reactive oxygen species (ROS) which are oxygen-containing chemical species [310]. Erlotinib, a cytostatic drug, is a chemotherapeutic drug for the treatment of pancreatic cancer and non-small cell lung cancer. The drug mainly functions by inhibition of epidermal growth factors (EGFR). Recently, a study was performed on human epithelial A431 cells with a combination of genistein and erlotinib. Genistein antagonized the Erlotinib-EGFR inhibitory effect, thereby effecting a different mechanism of cancer cell intrusion [311]. Another study demonstrated the combinatorial effect of genistein with sulforaphane on breast cancer MCF-7 and MDA-MB-231 cells. This combination resulted in an overall decrease in cell viability in both breast cancer cell lines thereby promoting cell death and cell cycle arrest in G1 phase (MCF-7 cells) and G2/M phase (MDA-MB-231 cells) [312]. A study in genistein-treated breast cancer MCF-7, and T47D cell lines also demonstrated the synergistic effect of genistein with cisplatin, paclitaxel, and tamoxifen chemotherapeutic drugs. As a result, in MCF-7 breast cancer cell lines, it was found that genistein + cisplatin and genistein + tamoxifen decreased the production of ROS and autophagy. Also, it enhanced the cell cycle at G2/M phase and decreased the cell cycle at the G_0_/G_1_ phase. On the contrary, this combination demonstrated a decrease in cell viability in T47D cell lines due to enhanced autophagic effect. Moreover, the genistein + tamoxifen combination led to an increase in cell viability in MCF-7 cell lines to a larger extent compared to genistein + paclitaxel combination [313].

Genistein also demonstrated synergistic action with resveratrol in the suppression of prostate cancer in the SV-40 Tag rat model. When this combination was fed to rats (high dose combination: 250 mg/kg AIN-76A diet and low dose combination: 83 mg genistein + 83 mg resveratrol/kg diet), there was a reduction in cell proliferation and reduced expression of insulin-like growth factor (IGF-1 factor) [314]. Many studies have shed light upon the poor bioavailability of genistein, quercetin, and biochanin A since their anti-cancerous activity such as tyrosine kinase activity, is hindered. A combinatorial approach can potentially overcome these programmed barriers. Prostate cancer LNCaP, DU-14, and PC-3 cells were subjected to treatment with genistein, quercetin and biochanin A. As a result, this combination demonstrated the inhibitory effect of tyrosine kinases, and also activated human aryl-hydrocarbon (ArH) receptors thereby inhibiting prostate carcinogenesis [315]. A combination treatment of genistein, quercetin, and EGCG was performed on prostate cancer CWR22Rv1 cells. Even though each of these polyphenols possesses non-overlapping activities, their combination led to the suppression of cell proliferation thereby altering the expression of androgen receptor, tumor suppressor *p53* and quinone reductase type 1(NQO1) enzyme [316].

### 5.4. Combinatorial Effect of Resveratrol with Other Polyphenols and Anti-Cancer Drugs

Resveratrol is a phytoalexin that can potentially counteract with many anti-cancerous properties. Due to its limited bioavailability, it can have hindrance in various molecular mechanisms associated with cancer. Studies have shown that resveratrol can overcome its bioavailability barriers when used in combination with other polyphenols and anti-cancer drugs. A study was conducted in breast cancer cell lines with the administration of a combination of resveratrol and thymoquinone. Thymoquinone is a phytochemical compound that possesses a large number of antioxidants, anti-inflammatory, anti-carcinogenic and chemo-sensitizing properties. In vivo, Balb/C mice were treated with resveratrol and thymoquinone, and as a result, there was an order of decrease in tumor size, followed by an increase in apoptosis, decrease in VEGF expression and inhibition of angiogenesis [317]. A study was conducted in prostate cancer cells, both in vitro and in vivo. Each of these polyphenols was administered alone or in combination in 22Rν1, DU145, and PC3 cell lines and in a TRAMP mice model. When resveratrol was combined with quercetin, the bioavailability of resveratrol was heightened by constraining its sulfation, thereby imparting higher anti-proliferation properties [318]. ADR is a hydrochloride salt, and a pegylated liposomal formulation. Numerous studies have shown that liposomal formulation is prone to increase the risk in cardiac events. Dexrazoxane, an FDA-approved drug, is effectively used to lessen ADR-induced cytotoxicity. However, the use of Dexrazoxane has led to interference with the efficacy with ADR, thereby increasing the risks of secondary tumors. Resveratrol, quercetin, curcumin, and ADR were administered alone and in combination in ovarian cancer ES2-Luc or A2780ADR cells which resulted in reducing ADR dozing via chemosensitization. This combination also resulted in tumor size reduction and enhanced apoptosis in ovarian cancer xenograft models [319].

Doxorubicin (DOX) is a chemotherapeutic drug which is primarily used against gastric cancer. Long-term exposure to doxorubicin in gastric cancer patients leads to the development of drug resistance and tumor regression. As a result, resveratrol reverses the Dox-resistance challenge by preventing EMT by controlling PTEN/*Akt* signaling pathways. A study was conducted in gastric cancer SGC_7901_ and MGC803 cell lines. A DOX-resistance gastric cancer cell line was developed by using a DOX concentration gradient method in SGC_7901_ cells. When these cells lines were subjected to treatment of resveratrol (RES) and doxorubicin, there was an enhanced cell survival of SGC_7901_ cells. A nude mice xenograft model was also used for the in vivo procedure where resveratrol and doxorubicin were administered alone and in combination. This combination enhanced the expression of caspase-9, increased the level of PTEN, TSC1, TSC2, and cleaved *caspase-3* and reduced p-Akt, p-mTOR, and p70 S6K significantly [320]. A few other combinations of resveratrol have also been investigated using in vivo models for their chemopreventive effects. A study was conducted with the potential abilities of resveratrol along with quercetin, apigenin, baicalein, curcumin, genistein and EGCG in vitro as well as in vivo. The study revealed that the combination of four out of six compounds: genistein, curcumin, EGCG, and resveratrol combination inhibited hedgehog signaling. Furthermore, the combination of apigenin, baicalein, and quercetin led to an overall decrease in *GLi1 mRNA* activity. When these compounds were fed in combination altogether, then there was an overall decrease in tumor size [321]. Gefitinib is a tyrosine kinase inhibitor (TKI) which interrupts signaling through various epidermal growth factors (EGFR) in target cells. A large number of clinical trials have demonstrated the potential benefits of gefitinib; however, like other anti-cancer drugs, its acquired resistance is a recurrent problem. Therefore, a potential combinatorial approach of dietary-based polyphenols with gefitinib might help to overcome the drug resistance. A combination study on grapes-based dietary polyphenols was conducted in vitro in breast cancer MDA-MB-231 cells as well as in vivo in a nude mice model. In vitro, the study revealed that resveratrol, quercetin, and catechin reduced Akt activity, induced the activation of AMPK, and inhibited mTOR signaling in breast cancer cell lines. Further, when resveratrol, quercetin and catechin were administered in combination with gefitinib, decreased gefitinib resistance occurred in these cell lines. In vivo, mice were fed with alone and in a combination 5 mg/kg of each resveratrol, quercetin, and catechin along with 200 mg/kg of gefitinib for 84 days. As a result, it was found that there was an inhibition of mammary tumor growth and metastasis to bone and liver in this mouse model [322]. A recent study was conducted in breast cancer patients wherein the patients were administered with a polyphenolic supplement consisting of a mixture of resveratrol (53.85 mg) and various plant extracts (orange: 53.85 mg, lemon: 53.85 mg, pomegranate: 161.5 mg, cocoa: 161.5 mg, olive: 161.5 mg, and grape seed: 53.85 mg). The components were blended and further encapsulated in hard gelatin capsules. Each patient (19 patients) was administered with 3 capsules since the beginning of the diagnosis until the night before the surgery and urine samples, blood samples, normal tissue samples and malignant tissue samples were collected. Upon metabolic profiling, a total of 101 metabolites were identified in urine, 69 metabolites were identified in plasma, 39 metabolites were identified in normal tissue and 33 metabolites were identified in malignant tissues. As a result, the metabolites identified in malignant tissues did not exhibit anti-proliferative activity or estrogenic estrogenic/anti-esterogenic activities in MCF-7 breast cancer cells [323].

### 5.5. Combinatorial Effect of Epigallocatechin Gallate (EGCG) with Other Polyphenols and Anti-Cancer Drugs

Sunitinib is a small molecule tyrosine kinase inhibitor, which is mainly used for the treatment of renal and pancreatic cancer. Due to the long-term administration of sunitinib, cancer patients are likely to develop drug resistance. This can be overcome by using sunitinib in combination with EGCG. A study was conducted in H460, MCF-7 and H1975 cell lines in vitro and in a xenograft mice model for in vivo study. When sunitinib was administered in combination with EGCG, the EGCG was seen to chemically interact with sunitinib thereby reducing its plasma concentration, leading to inhibition of various receptor kinases and downstream kinases, such as Erk1/2, STAT3 and phosphoinositide 3-kinase (*PI3K*)/*AKT* [324]. Furthermore, a study was conducted when EGCG was used in combination with vitexin-2-O-xyloside and raphasatin in breast cancer MDA-MB-231 and MCF-7 cell lines, and colorectal Caco-2 and LoVo cell lines. Vitexin-2-Oxyloside was extracted and further purified from seeds of *Beta vulgaris cicla*, and glucoraphasatin from *Raphanus sativus* L. This combination induced apoptosis through the mitochondrial pathway. Further analysis also revealed cell cycle arrest at the G0/G1 phase. This combination also controlled the activity of Bax, Bcl2, caspase-9, and ADP-ribose polymerase [325]. Another study was conducted in vivo and in vitro in lung cancer H1299 cell lines and CL3 mice wherein *N*-acetylcysteine and EGCG were administered in combination and alone. EGCG inhibited CL13 cell growth when used alone. However, when *N*-acetylcysteine (2 nM) was used in combination with EGCG, there was enhanced cell growth inhibition. This combination also increased ROS production and enhanced apoptotic activity [326].

The synergistic action of ECGC and pterostilbene in pancreatic cancer MIA PaCa-2 and PANC-1 cell lines was also revealed when administered in combination. In MIA PaCa-2 cells, this combination led to cell cycle arrest in S-phase arrest but not in PANC-1 cells. The combination also led to depolarization of mitochondria and upregulation of cytochrome- C in MIA PaCa-2 cells and not in PANC-1 cells. However, the increased apoptotic effect was observed in PANC-1 cells and not in MIA PaCa-2 cells. Therefore, this combination results in enhanced anti-cancerous activities of EGCG and pterostilbene when used in combination with each other [327]. In vitro study was conducted in pancreatic cancer MIA PaCa-2 cell lines. The study demonstrated the effect of EGCG and tumor necrosis factor-related apoptosis-inducing ligand (TRAIL) together on pancreatic cancer cells. This combination resulted in the reduction of cell proliferation, enhanced apoptosis and enhanced activation of caspase-8 activity. Hence, this combination could potentially serve as a potential therapeutic method for pancreatic cancer [328]. To further overcome the poor bioavailability of EGCG, another study was conducted in colorectal cancer in vivo and in vitro. Colorectal cancer HCT116 and SW480 cell lines were used, and cytotoxicity of both the compounds was measured individually and in combination. The study revealed that this combination of compounds led to minor enhancement in cytotoxicity. EGCG also induced enhanced apoptosis and cycle arrest in 5-fluorouracil-resistant colorectal cancer cells [329].

### 5.6. Combinatorial Effect of Sulforaphane with Other Polyphenols and Anti-Cancer Drugs

A large number of studies have demonstrated the synergistic effect of sulforaphane and green tea polyphenols (GTPs) in reactivating ERα expression in breast cancer MDA-MB-231 cell lines. A study was conducted in breast cancer MDA-MB-231 cell lines to investigate the consequence of ERα expression by reactivation of tumor suppressor genes (TSGs). The combination of sulforaphane and green tea polyphenols (GTPs) induced cell cycle arrest at G2/M phase by down-regulation of cell cycle regulatory proteins such as p21^CIP1/WAF1^ and KLOTHO that are mainly responsible for cell proliferation. Overall, this combination can induce the silencing of TSGs along with reactivation of ERα in MDA-MB-231 cell lines [330]. Withaferin A is isolated from winter cherry which is commonly found in India. A large number of studies have demonstrated the anti-cancerous effect of withaferin A which leads to reduced cell proliferation and cell viability in various cancer cell lines. A study was conducted in breast cancer MCF-7 and MDA-MB-231 cell lines to investigate the combinatorial impact of sulforaphane and withaferin A with their promising role in epigenetic gene expression of *DNMT1*, *DNMT3A*, *DNMT3B*, and *HDAC1*. It was detected that the combination had a synergistic effect on MCF-7 cells and an additive effect was observed on MDA-MB-231 cell lines thereby resulting in promotion of cell death as well as changes in BAX and BCL-2 activity. The combination also decreased HDAC expression and led to changes in DNMT1, DNMT3, and DNMT3B expression. The expression of DNMT1, DNMT3a, and DNMT3B was expressively reduced in MCF-7 and MDA-MB-231 cell lines [331]. The dietary polyphenols sulforaphane (SFN) and curcumin (CUR) have revealed tremendous chemopreventive effects in vivo and in vitro. However, the effects of these polyphenols are enhanced in different ways when used in combination. A study was conducted to investigate the combinatorial effect of SFN and CUR in a dose-dependent manner in liver cancer Hep-G2-C8 cell lines. Low doses of SFN; CUR; SFN + CUR enhanced the expression of *HO-1* and *UGT1A1* genes. Furthermore, higher dosage administration of SFN; CUR; SFN + CUR led to inhibition of cell viability [332].

Similar to other combinatorial approaches of polyphenols, EGCG has also demonstrated potential synergistic effects when administered along with SFN in vivo and in vitro in prostate cancer PC-3-AP-1 cell lines, ovarian cancer SKOV-ip1 and SKOVTR-ip2 cell lines and colon cancer HT-29-AP-1 cell lines. In prostate cancer, this combination resulted in down-regulation of the *Nrf2*, *ATF*, and *ELK-1* genes. The combination also inhibited SRF expression and CREB5 compared to individual dietary agents and caused the inhibition of SFN-induced expression of the *SLCO1B3* gene. In ovarian cancer, the combination of SFN and EGCG was administered in paclitaxel-sensitive SKOV-ip1 and paclitaxel-resistant SKOVTR-ip2 cell lines. This led to enhanced apoptosis in paclitaxel-resistant cells, increased expression of hTERT and DNMT1 in SKOVTR-ip2 cell lines and inhibition of cell viability in both the cell lines. In colon cancer HT-29 cell lines, low dose combination of SFN and EGCG enhanced AP-1 activity and decreased cell viability to 70%. And higher dose combination of SFN and EGCG decreased cell viability to 40% [333,334,335]. Many studies have demonstrated the anti-cancerous effects such as apoptosis and reduced tumor growth of acetazolamide (AZ) when used alone. The potential anti-cancerous properties of sulforaphane and AZ can be enhanced when used in combination. A study investigated the synergistic action of sulforaphane and AZ alone and in combination in a dose-dependent manner which led to the suppression of tumor growth, enhanced apoptosis and activation of caspase-3 and PARP activity. Furthermore, this combination also led to a significant effect on Ki-67, pHH3, cyclin D1 and down-regulation of p21 and p27 expression [336].

Another study demonstrated the combinatorial effect of sulforaphane, docetaxel, and paclitaxel in breast cancer SUM149 and SUM159 cell lines. Docetaxel and paclitaxel led to an increase in IL-6 and IL-8 secretion, and SFN caused a decrease in IL-6 and IL-8 secretion. However, when SFN was used in combination with docetaxel and paclitaxel, it was found that the IC50 of docetaxel and paclitaxel was reduced to 1.4 nM and 2.2 nM in SUM149 cells and 1.9 nM and 7.5nM in SUM149 cells. Therefore, this combination can potentially inhibit cell proliferation. An in vivo study was also performed to investigate the combined effect of SFN and docetaxel in a xenograft mouse model. As a result, the frequency of tumor formation was lower in combination when compared to SFN and docetaxel alone. Furthermore, the administration of SFN also reversed the enrichment of aldehyde dehydrogenase and reduced the size of mammosphere formation, which is caused by docetaxel and paclitaxel [337].

## 6. Conclusions

Traditional therapies such as chemotherapy and radiation are associated with substantial side effects. Therefore, it is imperative to develop novel approaches that have fewer side effects and are safer. A plethora of in vivo studies have demonstrated the use of dietary-based polyphenols in cancer prevention and therapy. Despite a limited number of in vitro studies and clinical trials showing the use of these polyphenols in cancer prevention and therapeutic measures against various molecular mechanisms and epigenetic modifications, these compounds portray a promising role in cancer prevention and therapy if used safely. Besides their promising roles in cancer treatment, polyphenols may possess a poor bioavailability when administered alone. However, the bioavailability and multiple preventive properties of these nutrients can be improved when administered in combination with other polyphenols, phytochemicals, and anti-cancer drugs. Therefore, future research directions can potentially expand upon the use of dietary-based polyphenols, especially in combinations, as a potent and effective method in cancer prevention and therapy.

## Figures and Tables

**Table 1:** Classification of polyphenols, chemical structure, molecular formula and their dietary source availability.

Polyphenols	Dietary Source	* Chemical Structure	** Molecular Formula	References
Apigenin	Grapefruit, parsley, onion, orange, tea and wheat sprouts	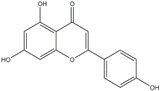	C_15_H_10_O_5_	[63]
Anacardic Acid	Cashew nuts	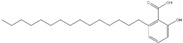	C_22_H_36_O_3_	[64]
Biochanin	Red clove, chickpea, clover sprout and kidney beans	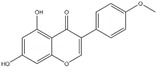	C_16_H_12_O_5_	[65,66,67]
Butein	*Rhus verniciflua, Caesalpinia sappan* and *Carthamus tinc-torius*	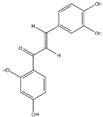	C_15_H_12_O_5_	[68,69]
Catechin	Green tea, apples, blackberries, dark chocolate and red wine	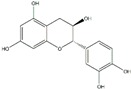	C_15_H_14_O_6_	[70]
Cyanidin	Acai berry, bilberry, blackberry, cranberry and raspberry	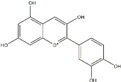	C_15_H_11_O_6_^+^	[71]
Curcumin (CUR)	Turmeric	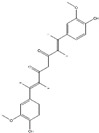	IC_21_H_20_O_6_ or C_21_H_20_O_6_	[72]
Caffeic Acid	Coffee and olive oil	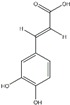	C_9_H_8_O_4_	[73]
Cholorogenic Acid	Pomegranate and berries	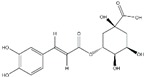	C_16_H_18_O_9_	[74]
Capsaicin	Chili peppers	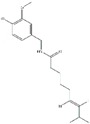	C_18_H_27_NO_3_	[75]
Daidzein	Soybeans and tofu	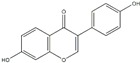	C_15_H_10_O_4_	[76,77]
Delphinidin	Cereal grains	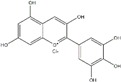	C_15_H_11_CIO_7_	[78]
Diosmetin	Vetch	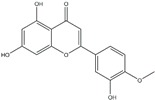	C_16_H_12_O_6_	[79]
Ellagic Acid	Blackberries, raspberries and pomegranate	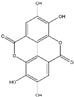	C_14_H_6_O_8_	[80]
Epicatechin	Milk, chocolates, and commercial reduced fat	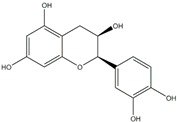	C_15_H_14_O_6_	[81]
Epigallocatechin-3-gallate (EGCG)	Green tea	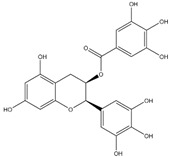	C_22_H_18_O_11_	[70]
Gallic Acid	Pomegranate, nuts and green tea	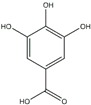	C_7_H_6_O_5_	[82]
Genistein	Fats, oils, beef, red clover, soybeans, and fava beans	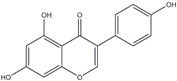	C_15_H_10_O_5_	[83,84]
Gnetol	*Gnetum ula, gnetum gnemon*, trees, shrubs and lianas	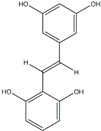	C_14_H_12_O_4_	[85]
Hesperidin	Bitter orange, petit grains, orange, lime and lemon	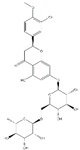	C_28_H_34_O_15_	[86]
Isoliquiritigenin	Rose petals	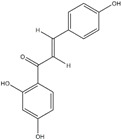	C_15_H_12_O_4_	[87]
Kaempferol	Apples, grapes, tomatoes, green tea, potatoes, onions and broccoli	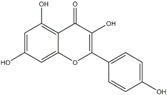	C_15_H_10_O_6_	[88]
Licochalcone A	Cranberry	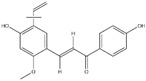	C_21_H_22_O_4_	[89]
Luteolin	Celery, broccoli, green pepper, parsley, thyme, dandelion, perilla and chamomile tea	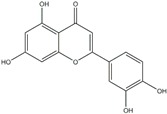	C_15_H_10_O_6_	[90,91]
Macluraxanthone	*Maclura tinctoria* (Hedge apple) and dyer’s mulberry	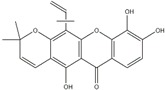	C_23_H_22_O_6_	[92]
Myricetin	Vegetables, fruits, nuts, berries, tea and red wine	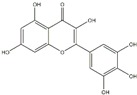	C_15_H_10_O_8_	[93]
Naringenin	Grapes	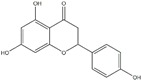	C_15_H_12_O_5_	[94]
Oxyresveratrol	*Morus alba* and *artocarpus lakoocha*	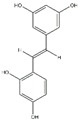	C_14_H_12_O_4_	[95]
Peonidin	Cranberries, blueberries, plums, cherries and sweet potatoes	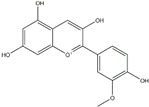	C_16_H_13_O_6_+	[96]
Piceatannol	Berries, grapes, rhubarb (*rheum*), passion fruit (*passiflora*) and white tea.	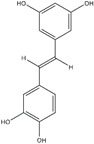	C_14_H_12_O_4_	[97]
Pterostilbene	Blueberries and grapes	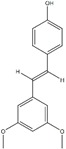	C_16_H_16_O_3_	[98]
Quercetin	Vegetables, fruits and beverages, spices, soups and fruit juices	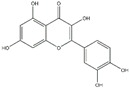	C_15_H_10_O_7_	[99,100]
Resveratrol	Almonds, blueberries and grapes	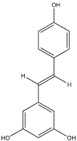	C_14_H_12_O_3_	[98]
Rosemarinic Acid	Rosemary	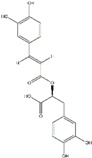	C_18_H_16_O_8_	[101]
Rutin	Citrus fruits, apple, berries and peaches	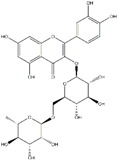	C_27_H_30_O_16_	[92,102,103]
Scopoletin	Vinegar, dandelion and coffee	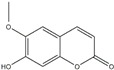	C_10_H_8_O_4_	[104]
Silibinin	Milk and artichokes	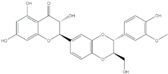	C_25_H_22_O_10_	[105,106]
Tangeretin	Citrus fruits	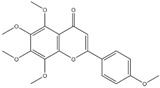	C_20_H_20_O_7_	[107]
Taxifolin	Vinegar	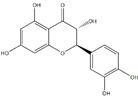	C_15_H_12_O_7_	[108]
Theaflavin	Tea leaves, black tea and oolong tea	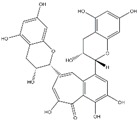	C_29_H_24_O_12_	[109]
Tricin	Rice bran and sugarcane	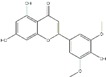	C_17_H_14_O_7_	[110]
Xanthohumol	Hop plants	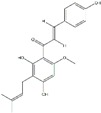	C_21_H_22_O_5_	[111]

* Chemical structures are drawn using ChemDraw software. ** Molecular formulas obtained through PubChem compound database.

**Table 2:** Assessment of polyphenols and their associated epigenetics modifications and molecular mechanisms (in vivo and in vitro studies) in cancer.

Dietary Compounds	Epigenetic Modifications	Gene Targets	* Overall Role in Cancer Progression	Dose	In Vitro Model	In Vivo Model	References
Apigenin	DNMT1 inhibitor DNMT3a inhibitor DNMT3b inhibitor HDAC1 inhibitor HDAC3 inhibitor	*NFE2L*, *NQO1, Nrf2*, *GRP78*, *GADD153, p21*, *waf1*, and *hTERT* *p53*	↓ Viability ↓ GLI1 expression Cell cycle arrest ↑ Apoptosis ↑ Caspase 3 Activity	20–30 μM 40–160 μM 20–50 μM 20–100 μM 20–40 μM	Pancreatic cancer Lung cancer (H460 cells) Breast cancer (BT-474 cells) Skin cancer (JB6 P+ cells) Prostate cancer (PC-3, 22Rv1 cells)	Mouse	[163,164,165,166,167,168,169,170]
Curcumin	DNMT inhibitor HAT inhibitor HDAC1 inhibitor Down-regulation of histone methylation	*CDKN2B, NEUROG1, NFE2L2, Nrf2, Neurog1 RASSF1A, p16, SPARC, SOCS1, SOCS3, p53, p21, GAS5, HOTAIR, H19, AF086415, AK095147, RP1-17916.3, MUDENG, AK056098, AK294004*	↓ Proliferation ↑ Apoptosis ↓ ERK, MKK4, JNK activity ↓ Bcl-2 ↓ Akt expression ↑ p38 activation ↓ Cell viability ↑ Bax Activity	40 μM 0–100 μM 0–50 μM 2.5–160 μM 7.5–10 µM 0–50 μM	Breast cancer (MCF-7 cells) Prostate cancer (LnCap cells) Colorectal cancer (HCT116, HT29 and RKO cell lines) Gastric cancer (MiaPaCa-2, PANC-1 cells) Breast cancer (MCF-7 cells) Ovarian cancer (SKOV3 cells)		[171,172,173,174,175,176,177,178,179,180,181,182]
Daidzein	DNMT inhibitor HDAC inhibitor	*BRCA1, GSTP1, EPHB2, MMP-2, BRF1, BRF2, RARβ*	↓ Proliferation ↑ Apoptosis ↓ ERK, MKK4, JNK activity	200–600 μM 20–100 μM 3–50 μM 12.8–100 μM	Colorectal cancer Breast cancer (MDA-MB-231 cells) Liver cancer (SKHEP-1 cells) Prostate cancer		[62,183,184]
Delphinidin	DNMT inhibitor HAT inhibitor HDAC-3 inhibitor	*p21*, *WAF1/Cip1, p53, p27/KIP1*	↓ Cell Proliferation ↓ Metastasis Cell Cycle Arrest Oxidative Stress	3–90 μM 30–240 μM 5–60 µM 100 μM	Prostate cancer (PC3 cells) Colorectal cancer (HCT116 cells) Lung cancer (NCI-H441 cells) Prostate cancer (LNCaP cells)	Athymic nude mice	[185,186,187,188]
Myricetin	DNMT inhibitor Increased SIRT1 activity	*GSTP1, RARβ, HIN-1*	↑ Apoptosis Autophagy	20–40 μM 5–25 μM 100 μM	Gastric cancer (GC HGC-27, SGC7901 cells) Breast cancer Colon cancer (HCT-15 cells)		[189,190,191]
Ellagic Acid	DNMT1 inhibitor DNMT3b inhibitor HDAC inhibitor	*p16INK4a*, *RASSF1A*, *GSTP1*, *HIN1, VEGF*, *MMP-2*, *p53*	↑ Apoptosis Cell proliferation Cell migration ↑ Caspase 3 Activity ↑ Caspase 9 Activity	50–200 μM 10–100 μM	Colorectal cancer Prostate cancer (PC-3 cells) Breast cancer (MCF-7 cells)		[192,193,194,195]
EGCG	DNMT inhibitor HAT inhibitor Down-regulation of histone methylation Effect on histone ubiquitination Upregulation of histone phosphorylation	*p16INK4a; RARβ; MGMT; hMLH1; GSTP1; WIF-1; RECK*, *Cip1/p21* *AT102202*, *p53, p21*	↓ Invasiveness ↓ Proliferation ↑ Apoptosis ↑ Caspase 3 Activity ↑ Caspase 8 Activity ↑ Cytochrome c	1–40 μM 1–50 μM 5–20 μM 20–100 μM 1–50 μM 0–20 μg/mL	Breast cancer (MCF-7 cells) Colorectal cancer (HT-29 cells) Lung cancer (CL1-5 cells) Gastric cancer (MKN-1, MKN-28, MKN-45, NUGC-3 and TMK-1) Colorectal cancer Skin cancer (A431 cells)	Xenograft mice	[47,196,197,198,199,200,201,202,203,204,205,206,207,208]
Hesperidin	DNMT inhibitor HDAC inhibitor	*GSTP1, Akt, LAMTOR2, LAMTOR3, LAMTOR5, MAPK1, KRAS, HRAS, MAPK3*	↓ Cell proliferation ↑ Apoptosis ↑ Glucose uptake ↑ ASK1/JNK pathway ↑ ROS production	40–90 μM 40–200 μM 650 μM 20–50 μM 90 μM 50 μM	Breast cancer (MCF-7, MDA-MB-231 Cells) Liver cancer Cervical cancer (SiHa cells) Esophageal cancer Prostate cancer (PC-3 cells) Endometrial carcinoma (ECC-1 cells)	Xenograft mice Rats	[209,210,211,212,213,214,215]
Kaempferol	DNMT3a inhibitor DNMT3b inhibitor HDAC1 inhibitor	*p-Akt, ERK, MSK1, CD1, p23, BTG3, BRCA1, MGMT*, and *hMLH1*	↑ Apoptosis ↓ Glucose uptake Autophagy Cell cycle arrest	100 μM 10–50 μM 0–60 μM 4 µM 50 μM 20 mg/kg	Liver cancer (SK-HEP-1 cells) Lung cancer (A549 cells) Colorectal cancer (HT-29 cells) Breast cancer (MCF-7 cells) Gastric cancer (G9a cells) Gastric cancer (MKN28, SGC7901 and GSE-1 cells)	Athymic mice Xenograft mice	[216,217,218,219,220,221]
Luteolin	DNMT inhibitor HDAC inhibitor	*VRK1, MPK2*	↑ Apoptosis Cell cycle arrest Cell invasion	20–50 μM 5–50 μM 10–40 μM 20–100 μM 10 μM	Esophageal cancer Lung cancer (A549 cells) Breast cancer (MCF-7 cells) Colorectal cancer Lung cancer (A549 cells)	Xenograft Mice	[222,223,224,225,226,227]
Pterostilbene	DNMT inhibitor Decreased SIRT1 activity	*p53, NF-κB* and miRNA488	↑ Apoptosis Cell cycle arrest	25–75 μM	Breast cancer (MCF-7 and MDA-MB-231 cells)	Mice	[137,228,229,230,231,232]
Polyphenol- rich Strawberry extract (PRSE)		*Csf1, Mcam, Nr4a3*, *SET, Gpnmb, Itgb3*, *CC17, Ctsl, Cxcr4, Htatip2, Mmp-10* and *Mmp3*	↓ Cellular Viability ↓ Number of cells in S phase Accumulation of cells in G1 phase ↓ Tumor Weight ↓ Tumor Volume	0.5–5 mg/mL	Breast Cancer (MCF-7 and A-17 cells)	Mice	[233]
Genistein	DNMT1 inhibitor DNMT3a inhibitor DNMT3b inhibitor HDAC inhibitor HAT activator Decreased SIRT activity Upregulation of histone methylation	*p16INK4a; RAR β; MGMT; PTEN; CYLD, MGMT, CDKN2A, BTG3, TERT, GSTP1, EZH 2*, *FoxM1, sFRP1, p21, p16, PTEN, CCLD, p53, FOXA3, SIRT1, BTG3, hTERT, RAR, HOTAIR*	↓ Proliferation ↓ Tumorigenesis ↑ Apoptosis ↑ mRNA expression of tumor suppressor genes ↑ H2A variant at serine 139 (γ-H2AX)	25–75 μM 0.5–50 μM 0.5–50 μM 100 μM 20–50 μmol/L 5–100 μM	Lung cancer (H446 cells) Breast cancer (MCF-7, MDA-MB-231 cell lines) Prostate cancer (LAPC-4 cells) Cervical cancer Esophageal squamous cell carcinoma Prostate, breast cancer and renal cancer	Agouti mice Sprague-Dawley rats	[234,235,236,237,238,239,240,241,242]
Gallic Acid	DNMT1 inhibitor DNMT3b inhibitor	*MMP-2, MMP-9, ADAM17, Erk/p-Erk, p-Akt*	↑ Apoptosis	200 μM 80.5 μM 25–200 μM 0–40 μg/mL 50 μM	Colorectal cancer Breast cancer (MCF-7 cells) Prostate cancer (PC-3 cells) Cervical cancer (HeLa and HTB-35 cells) Oral cancer (H1299 cells)		[243,244,245,246,247]
Naringenin	DNMT1 inhibitor DNMT3a inhibitor DNMT3b inhibitor HDAC1 inhibitor	*ATF3, PARP, p38, MMP-9, ERK, Akt*	↑ Apoptosis Cell cycle arrest ↓ Cell growth ↓ Cell proliferation	100 M μM 20–80 μM 20 or 50 μmol/L 25–200 μM 50–100 μM	Lung cancer (A549 cells) Gastric cancer Esophageal cancer (KYSE-510 cells) Liver cancer (HepG2, Huh-7, and HA22T cells) Colorectal cancer (HCT116, SW480, Lovo, and HT-29 cells)	Resection mice Rats	[248,249,250,251]
Piceatannol	DNMT3a inhibitor Decreased SIRT1 activity	*RASSF1A*, *GSTP1*, *HIN1 mTOR*	↑ Apoptosis Cell cycle arrest	30 μM 25 μM 50 μM	Colorectal cancer (HCT116 and HT29 cells) Prostate cancer (DU145 and PC-3 cells) Breast cancer (MCF-7 cells)	Mice	[193,252,253,254]
Quercetin	DNMT1 inhibitor HDAC inhibitor Down-regulation of histone demethylation	*CDKN2A (p16INK4a)*, *AMPK, Akt*, *DBH-AS1, p53*	↓ Proliferation ↓ Akt phosphorylation ↓ Angiogenesis ↑ Caspase 3 and 7 activity ↑ Bax Activity	1–200 μM 40–160 μM	Liver cancer (HepG2 and SMCC-7721 cells) Lung cancer (A549 cells) Gastric cancer (AGS and MKN28 cells) Colon cancer (HCT116 cells)	Mice	[47,255,256,257,258,259,260,261,262]
Xanthohumol	DNMT inhibitor HDAC inhibitor	*CXCR4, CXCL12, p53*	↑ Apoptosis Cell cycle arrest Cell Invasion Cell proliferation Cell migration	14–42 μM 5–40 μM	Lung cancer (A549 cells) Liver cancer Breast cancer Prostate cancer (DU145 and PC3 cells)	Transgenic Mice	[263,264,265,266,267]
Sulforaphane	DNMT3a inhibitor DNMT3b inhibitor HDAC inhibitor Upregulation of histone phosphorylation	*NFE2L2, TERT, Nrf2, ZEB1, COX-2/MMP-2, 9/snail, p21, p27, RBP2*	↓ Proliferation ↑ Apoptosis Cell cycle arrest	50 μM 5–10 µM 0–30 μM	Colorectal cancer Breast cancer (MDA-MB-231 and MCF-7 cells) Bladder cancer (T24 cells and 5637 cells)	Xenograft Mice	[268,269,270,271,272,273,274,275]
Resveratrol	DNMT3a inhibitor DNMT3b inhibitor Decreased SIRT1, SIRT2, and SIRT3 activity HAT inhibitor Regulation of histone phosphorylation	*PTEN, XRCC1, p21 p16, MDR1, SP-1, STIM1, FOXO, PCGEM1, PRNCR1, PCAT29, AK001796, MALAT1, u-Eleanor, LINC00978, p53, p21*	↓ Proliferation ↑ Apoptosis ↓ Metastasis ↑ Caspase 8/9 activity ↑ Bax Activity ↓Bcl-2 Activity	50–150 μM 5–50 μM 50–200 μM 20–150 μM 25–100 μM 150–250 μM 25–100 μM	Breast cancer (MCF-7 cells) Lung cancer (H1703 and H1975 cells) Gastric cancer (Ki67 cells) Colon cancer (HT-29 cells, COLO 21 cells) Prostate cancer (PC3 and DU145 cells) Cervical cancer Liver cancer (Huh7 cells)	Xenograft Mice	[179,276,277,278,279,280,281,282,283,284,285,286,287,288]

*↓- decreased, ↑- increased.

**Table 3:** Impact of combinatorial therapy (polyphenols, phytochemicals, and anti-cancer drugs) on epigenetic modifications and molecular mechanisms (in vivo and in vitro studies) in cancer.

Combinatorial Therapy	Organ of Study	In Vitro Model		In Vivo Model		*Epigenetic Modifications and Molecular Mechanism	References
		Cell Lines	Dose				
Apigenin + IFNγ	Cervical cancer	HeLa and SiHa cells	5–15 μM + 100 ng/mL			↓ Cell viability ↑ Apoptosis Up-regulation of *DNMT1*	[289]
Apigenin + Paclitaxel	Ovarian cancer	HeLa, A549, Hep3B and HEK293A cells	15 µM + 4 nM			Apoptosis through suppressing SOC activity ↑ ROS and caspase-2 cleavage.	[290]
Apigenin + Cisplatin	Renal cancer	Human renal proximal tubular epithelial (HK-2) cells	5–20 µM + 40 µM			Apigenin reduced cisplatin-induced caspase-3 activity and PARP cleavage ↓ ROS production and p53 activation *Akt* phosphorylation	[293]
	Prostate cancer	PC3 PCa cells	15 μM + 7.5 µM			Upregulation of Caspase-8, Apaf-1 and p53 Down-regulation of Snail expression. Repressed phosphorylation of p-PI3K and p-Akt	[292]
Apigenin + doxorubicin	Lymphoid leukemia	CCRF, CEM, Jurkat and THP-1 cells	0.01 μM + 0.4 μM			↑ caspase-3 activity Cell cycle arrest at S and G2/M phase ↑ DNA damage	[294]
Apigenin + etoposide						↑ *caspase-3* activity ↑ *caspase-8* activity ↑ *caspase-9* activity	[294]
Apigenin + Naringenin	Colon cancer			Mice	0.1 μM + 0.05 μM	↑ ER-mediated YAMC cell growth ↑ activation of ERβ	[295]
Apigenin + Gemcitabine	Pancreatic cancer	MiaPaca-2, AsPC-1 cell lines	30 μM + 05–2 μM	Xenograft mice model		Down-regulation of NF-κB activity Suppression of Akt activation	[296]
Curcumin + Resveratrol	Prostate cancer	PTEN-CaP8 cancer cells		B6C3F1/J mice		↓ p-*Akt*, and cyclin D1 activity	[297]
Curcumin + Catechins	Colon cancer			DMH rat model	0.1% + 0.1%	↑ Apoptotic index ↓ Proliferation index	[298]
Curcumin + Piperine	Breast cancer	MCF-7 and Sum159 cells	5–25 μM + 5–25 µM			Inhibit mammosphere formation ↓ stem cell self-renewal ↓ in the cell percentage expressing stem cell marker ALDH1 Inhibit Wnt Signaling	[300]
Curcumin + Paclitaxel	Breast cancer	MDA-MB-231 cells	10 μM + 10 µM	Nude mice model	100 mg/kg + 7 mg/kg	Inhibition of tumor cell growth ↓ Tumor size ↓ Tumor cell proliferation ↑ Expression of *MMP-9*	[301]
Curcumin + Arctigenin + Green tea + Epigallocatechin gallate (EGCG)	Prostate cancer	LNCaP cells	5–10 μM + 1 μM + 40 μM			↑ Apoptosis ↑ Cell cycle arrest at G0/G1 phase ↓ Activation of *NFκB*, *PI3K*/*Akt* and Stat3 pathways ↓ Cell migration	[303]
	Breast cancer	MCF-7 cells	5–10 μM + 1 μM + 40 μM				
Curcumin + Epigallocatechin gallate (EGCG)	Non-small lung cancer	A549 and NCI-H460 cells	10 μmol/L +10 μmol/L			↓ Tumor size Cell cycle arrest at G1 and S/G2 phase Inhibition of expression of cyclin B1 and cyclin D1 Inhibition of clonal formation Down-regulation of DNMTs	[304,305]
	Prostate cancer	LNCaP, DU145 and PC3 cells	50 μM + 100 µM			↑ p21 expression Cell cycle arrest at S, G2/M phase	
Curcumin + Dasatinib	Colon cancer	HCT-116, HT-29 and SW-620		APC^Min+/−^ mice		↓ Cell proliferation ↑ Apoptosis ↑ Invasion through the extracellular matrix ↑ Tubule formation by endothelial cells	[306]
Curcumin + TRAIL	Prostate cancer	LNCaP cells		PC3 cells		↑ Apoptosis ↑ MMP-9, MMP-2, caspase-3, and caspase-9 activity	[307,308]
Curcumin + Gemcitabine	Pancreatic cancer			BxPC3, MiaPaCa2 and Panc1 PDAC cells	100 mg/kg + 25 mg/kg	↓ Tumor growth ↓ *NF-κB* activity ↓ VFGF expression	[309]
Genistein + Delphinidin + Alternariol (AOH)	Colon cancer	HT-29 cells	25 μM + 100 μM + 50 μM			↑ Cytotoxic effect ↑ Genotoxicity effect ↑ Topoisomerase poisoning ↓ ROS generation	[310]
Genistein + Erlotinib	Bladder cancer	A431 cells	100 μM + 10 nM			Inhibitor of EGFRs Overexpression of RTKs	[311]
Genistein + Sulforaphane	Breast cancer	MCF-7 and MDA-MB-231 cells	5 µM + 10-15 µM			Cell cycle arrest at G1 and G2/M phase ↓ Cell viability ↑ Apoptosis	[312]
Genistein + Cisplatin	Breast cancer	MCF-7 and T47D cells	1 μM + 10 μM			↓ ROS production ↑ Cell viability ↓ Autophagy ↓ Apoptosis Cell cycle arrest at subG_0_/G_1_ phase	[313]
Genistein + Tamoxifen			1 μM + 10 μM				
Genistein + Paclitaxel			1 μM + 10 μM				
Genistein + Resveratrol	Prostate cancer			Rats	83 mg/kg + 83 mg/kg	↓ Tumor growth Inhibition of Growth factors	[314]
Genistein + Quercetin + Biochanin A	Prostate cancer	PC-3, LNCaP, DU-145 cells	8.33 μM + 8.33 μM + 8.33 μM			*↑* BAX/BCL-2 activity ↑ caspase-3 activity ↑ ER-β activity ↑ p-JNK activity ↓ p-ERK activity ↓ PCNA activity ↓ Cell proliferation ↑ Apoptosis	[315]
Genistein + EGCG + quercetin	Prostate cancer	CWR22Rv1 cells	2.5 μM + 2.5 μM + 2.5 μM			↑ *p53* activity ↓cell proliferation	[316]
Resveratrol + Thymoquinone	Breast cancer	MCF-7 and T47D cells	10 μM + 25–300 μM	Balb/C mice	50 mg/kg + 50 mg/kg per day	↑ Apoptosis, ↓ Tumor growth Inhibition of angiogenesis	[317]
Resveratrol + Quercetin	Prostate cancer	22Rν1, DU145 and PC3 cells		TRAMP mice model	625 mg/kg + 60 mg/kg	↓ Cell proliferation	[318]
Resveratrol + Curcumin + ADR	Ovarian cancer	A2780 cells	10 μM +2 μM +1 μM	Xenograft model in Athymic mice	19.68 mg/kg + 26.06 mg/kg + 5mg/kg	↓ Cell viability ↓ Tumor size ↑ Apoptosis	[319]
Resveratrol + Quercetin + ADR	Ovarian cancer	A2780 cells	10 μM +10 μM +1 μM	Xenograft model in Athymic mice	19.68 mg/kg + 5.2 mg/kg + 5mg/kg	↓ Cell viability ↓ Tumor size ↑ Apoptosis	[319]
Resveratrol + Doxorubicin	Gastric cancer	SGC_7901_ and MGC803 cell lines	50 mg/L + 0.75 mg/L	Nude xenograft mice model	3 mg/kg + 50 mg/kg$	↑ expression of caspase 9 ↑ PTEN, TSC1, TSC2, and cleaved caspase 3 ↓ p-*AkT*, and mTOR activity	[320]
Resveratrol + Genistein + Quercetin + Apigenin + Baicalein + Curcumin + EGCG	Prostate cancer	PC3 and LNCaP cells		TRAMP mice model	1 μmol/L + 20 μmol/l + 10 μmol/L + 3 μmol/L+ 3 μmol/L+ 10 μmol/L+ 10 μmol/L	**↓** GLi1 mRNA activity$**↓** Tumor size	[321]
Resveratrol + Pomegranate + Orange + Lemon + Olive + Cocoa + Grape seed	Breast Cancer	MCF-7 cells			53.85 mg + 161.5 mg + 53.85mg + 53.85 mg + 161.5 mg + 161.5 mg + 53.85 mg	↓ Anti-proliferative activity $↓ Estrogenic estrogenic/anti-esterogenic activity	[323]
EGCG + Sunitinib	Breast cancer Non-small cell lung cancer	H460, H1975, and MCF-7 cells	50 μM + 3 μM	Xenograft mice model	50 mg/kg + 40 mg/kg	Suppression of IRS/MAPK/p-S6K1 signaling	[324]
Resveratrol + Quercetin + Catechin + Gefitinib	Breast cancer	MDA-MB-231 cells	15 μM + 15 μM+ 15 μM + 15 μM	SCID mice model	5 mg/kg +5 mg/kg + 5 mg/kg + 5 mg/kg + 200 mg/kg	↓Tumor size Cell cycle arrest at S phase ↓ Cell viability Inhibition of Akt/mTOR signaling	[322]
EGCG + Vitexin-2-O-xyloside + Glucoraphasatin	Breast cancer	MDA-MB-231 cells MCF-7 cells	(1980 ± 94) μg/mL + (1200 ± 66) μg/mL + (21 ± 6) μg/mL + (350 ± 47) μg/mL + (350 ± 48) μg/mL + (31 ± 4) μg/mL			Induction of apoptosis Cell cycle arrest at Regulation of Bcl2, Bax, cleaved caspase-9 and PARP ↑ ROS production	[325]
	Colorectal cancer	Caco-2 cells LoVo cell	(21 ± 3) μg/mL + (120 ± 9) μg/mL + (16 ± 4) μg/mL + (135 ± 16) μg/mL + (158 ± 13) μg/mL + (36 ± 5) μg/mL				
EGCG + NAC	Lung cancer	H1299 cells	100 μM + 2 mM	CL13 mice		↑ Apoptosis ↑ ROS production	[326]
EGCG + Pterostilbene	Pancreatic cancer	PANC-1 and MIA-Pa-Ca-2 cells	20–40 μM + 30 μM			↓ Cell proliferation Cell cycle arrest at S phase ↑ caspase-3/7 activity	[327]
EGCG + TRAIL	Pancreatic cancer	MIA-Pa-Ca-2 cells	50 μg/mL + 5 ng/mL			↑ Apoptosis ↑ Activation of caspase-8 and caspase-9	[328]
EGCG + 5- Fluorouracil	Colorectal cancer	HCT-116 and SW480 cells	25–400 μM + 2.5–40 μM	Xenograft mice model		↓ miR-34a, miR-145, and miR-200c Cell cycle arrest ↓ *Notch1*, Bmi1, Suz12, and *Ezh2* activity	[329]
EGCG + 5- Fluorouracil	Colorectal cancer	HCT-116 and SW480 cells	25–400 μM + 2.5–40 μM	Xenograft mice model		↓ miR-34a, miR-145, and miR-200c ↑ spheroid formation ↓ *Notch1*, Bmi1, Suz12, and Ezh2 activity Cell cycle arrest at G0/G1 phase	[329]
Sulforaphane + Green tea polyphenols (GTPs)	Breast cancer	MDA-MB-231 cells	5–10 μM + 20 µg/mL			Reactivation of Tumor suppressor genes (TSGs) *p21^CIP1/WAF1^* and *KLOTHO* Cell cycle arrest at G2/M phase ↓ CDK1 and CDC25C expressions Inhibition of IGF-1 pathways	[330]
Sulforaphane + Withaferin-A	Breast cancer	MCF-7 and MDA-MB-231 cells	5 μM + 10 μM			↓ *HDAC1* Inhibition of *DNMT1*, *DNMT3A*, and *DNMT3B* ↑ Apoptosis ↓ BAX/BCL-2 activity	[331]
Sulforaphane + Curcumin	Liver cancer	HepG2-C8 cells	Low dose: 12.5 μM + 10 μM High dose: 50 μM + 25 μM			↑ ARE-luciferase activity ↑ Expression of *HO-1* and *UGT1A1* ↑ *Nrf2* mRNA levels	[332]
Sulforaphane + EGCG	Prostate cancer	PC-3-AP-1 cells	Low Dose: 25 μmol/L +20 μmol/L High Dose: 25 μmol/L +μmol/L	*Nrf2*-deficient mice	45 mg/kg + 100 mg/kg	Inhibition of AP-1 activity Down-regulation of Nrf2-dependent genes	[334]
	Ovarian cancer	SKOV-ip1 and SKOVTR-ip2 cells	10 μM + 20 μM			↑ Expression of *hTERT*, *DNMT1* ↓ Cell viability Cell cycle arrest in G_2_/M and S phases ↑ Apoptosis	[333]
	Colon cancer	HT-29 cells	Low Dose: 25 μM + 20 μM High Dose: 10 μM + 20 μM			↓ Cell viability ↑ AP-1 activity	[335]
Sulforaphane + Acetazolamide (AZ)	Urothelial cancer	HTB-9 and RT112(H) cells	40 μM + 40 μM			↑ Apoptosis ↓ Ki-67, pHH3, cyclin D1 activity Cell cycle arrest Inhibition of *Akt* kinase activity Downregulation of p-*Akt* (Ser473) and *p-S6* activity	[336]
Sulforaphane + Docetaxel + Paclitaxel	Breast cancer	SUM149 and SUM159 cells	5 μM + 0–20 μM	Xenograft mice model	50 mg/kg daily + 10 mg/kg weekly	↓ Tumor growth Inhibition of NF-κB p65 translocation	

↓- decreased, ↑ - increased.

## Abbreviations

AOH	Alternariol
AZ	Acetazolamide
Bp	Base pair
CUR	Curcumin
DMH	1, 2-dimethylhydrazine
DOX	Doxorubicin
DNMTs	DNA methyltransferases
DNMT1	DNA methyltransferase 1
DNMT3a	DNA methyltransferase 3 Alpha
DNMT3b	DNA methyltransferase 3 Beta
EGFR	Epidermal growth factors
EGCG	Epigallocatechin-3-gallate
GTPs	Green tea polyphenols
HAT	Histone acetyltransferase
HDAC	Histone deacetylase
HMT	Histone methyltransferase
HDM	Histone demethylase
H3-K27	Histone H3 on lysine 27
H3-K9	Histone H3 on lysine 9
IFNγ	Interferon γ
miRNA	microRNA
NSCLC	Non-small cell lung cancer
PI	Proliferation index
PRSE	Polyphenol- rich Strawberry extract
ROS	Reactive oxygen species
*SAM*	*S*-adenosyl-L-methionine
SNP	Single Nucleotide Polymorphism
SFN	Sulforaphane
miRNAs	microRNAs
NAC	*N*-acetylcysteine
NQO1	Quinone reductase type 1
PTMs	Post-translational modifications
TSGs	Tumor suppressor genes
TRAIL	Tumor necrosis factor genes
YMAC	Young adult mouse colonocytes cells
